# Current Advances in Diagnosis, Therapeutics, and Surgical Interventions for the Management of Refractory Gastroesophageal Reflux Disease (GERD): An Update

**DOI:** 10.7759/cureus.69001

**Published:** 2024-09-09

**Authors:** Bhawana Ganesh Shashi, Shaik N Hafsa

**Affiliations:** 1 General Medicine, Divisional Hospital, Delhi, IND; 2 Otorhinolaryngology, Lady Hardinge Medical College, New Delhi, IND; 3 Otorhinolaryngology, Rajiv Gandhi University of Health Sciences, Bengaluru, IND

**Keywords:** acid reflux, erosive esophagitis, esophageal disorder, gastroesophageal reflux disease (gerd), laryngopharyngeal reflux disease, non-erosive reflux disease, refractory to ppi

## Abstract

Gastroesophageal reflux disease (GERD) is a chronic illness characterized by complications arising from the reflux of stomach contents, which significantly lower the quality of life, increase morbidity, and increase medical expenses associated with treating the condition. The main goal of treatment in GERD is symptomatic relief, relapse prevention, and healing of erosive esophagitis. The treatment mainly involves lifestyle changes to control acid production and proton pump inhibitors (PPIs) as the first line of treatment. Endoscopic interventions or anti-reflux surgery may be beneficial in relieving symptoms in people whose symptoms are triggered by reflux. In this review, we discuss the pathophysiology and newer diagnostic and treatment modalities including available surgical management options to manage refractory GERD.

## Introduction and background

Gastroesophageal reflux disease (GERD) manifests as the regurgitation of stomach contents into the esophagus, leading to esophageal damage and potential complications. GERD, which affects 10-20% of adults in Western countries, has a major influence on day-to-day living. Increased morbidity and a notable decline in health-related quality of life are linked to the disease, along with everyday discomfort, frequent doctor visits, pathological testing, and long-term care. All these lead to higher direct and indirect costs of living [1].

Long-standing disease in patients may lead to dysphagia, esophagitis, Barrett's esophagus, and esophageal cancer. GERD results from a failure of the anti-reflux barrier, i.e., the diaphragmatic crura, and lower esophageal sphincter (LES) due to transient lower esophageal sphincter relaxation (TLESR) reducers, which occur after swallowing among 90% of reflux episodes. Prolonged TLESRs often lead to esophageal injury, while other possible causes of refractory GERD (rGERD) may include weakly acid reflux, duodenogastroesophageal/bile reflux, comorbidities, heterogeneity of genotypes, visceral hypersensitivity, delayed gastric emptying, psychiatric comorbidity, and excessive functional bowel disorder [2]. Proton pump inhibitors (PPIs) are a class of drugs that suppress acid production and are commonly used to treat heartburn. Despite the significant efficacy of PPIs, as many as 30% of patients with rGERD fail to respond to PPI therapy, leading to persistent symptoms. rGERD has been defined as having typical reflux symptoms persistent for at least 8-12 weeks and does not improve with double-dose PPI medication [3]. This review examines and summarizes the currently available data on rGERD, including the diagnostic and therapeutic options to manage it.

## Review

Etiology and pathophysiology of the disorder: an overview

The pathophysiology of GERD is still not fully understood, but sliding hiatus hernia (HH), LES pressure, TLESR, the acid pocket, obesity, increased distensibility of the esophagogastric junction (EGJ), prolonged esophageal clearance, and delayed gastric emptying have been implicated in the induction or augmentation of reflux. Additionally, the acidity of the refluxate, its proximal extent, the gaseous nature of the refluxate, the occurrence of duodenogastroesophageal reflux, longitudinal muscle contraction, mucosal integrity, and peripheral and central sensitization influence the precipitation and augmentation of rGERD. Knowledge of the etiology and pathophysiology of rGERD is essential to learn from the past and to translate this knowledge into novel therapeutic principles [4]. The causative factors of persistent rGERD also include poor drug compliance and improper dosing of PPI, esophageal hypersensitivity, nocturnal acid breakthrough (NAB), PPI metabolism, and CYP2C19 polymorphism. On the other hand, a myriad of alternative diagnoses such as Zollinger-Ellison syndrome, autoimmune skin conditions (e.g., lichen planus), pill-induced esophagitis, infectious esophagitis, caustic esophagitis, radiation-induced esophagitis, eosinophilic esophagitis, esophageal cancer, achalasia, gastroparesis, *Helicobacter pylori* carrier status, and rumination syndrome contribute towards non-reflux-related GERD [5].

Diagnosis of rGERD symptoms

Comprehensive clinical assessment, upper gastrointestinal (GI) endoscopy, and high-definition manometry are employed for assessing esophageal motility and reflux monitoring, ideally with a multichannel intraluminal pH-impedance monitor. Since there is no known "gold standard" test for the confirmatory diagnosis of GERD, it must be empirically diagnosed and treated in all cases presenting with typical symptoms like heartburn and regurgitation. It is important to note that a diagnosis of GERD based solely on symptoms is not very accurate when compared to objective evidence of the disease determined by upper endoscopy and esophageal pH monitoring [6]. Several questionnaires have been formulated to enhance the clinical diagnosis of GERD. These questionnaires incorporate certain symptoms and quality-of-life scales to assess the condition of the patient. The most popular questionnaires employed for this purpose are the Reflux Disease Questionnaire (RDQ) and the Gastroesophageal Reflux Disease Questionnaire (GERDQ). The GERDQ can be used to identify heartburn and regurgitation, which are common symptoms of GERD. The RDQ has been revised to include negative predictors related to nausea and epigastric discomfort, as well as positive forecasters involving heartburn and regurgitation. Rey et al. applied many scores including RDQ and GERDQ on 4574 patients included in a multicenter study and found that an RDQ score of more than 3 had good sensitivity (65%) and specificity (71%) for differentiating troublesome and non-troublesome symptoms of GERD and it was comparable to the effectiveness of a gastroenterologist's clinical judgment [7]. The same problems apply to surveys intended to gather extraesophageal symptoms, such as persistent cough, hoarseness, asthma, etc., that may be indicative of rGERD [8]. Although these questionnaires are effective in clinical trials, they are not often commonly utilized in day-to-day practice.

Esophagogastroduodenoscopy (EGD)

An upper endoscopy exam called EGD entails a quick procedure to look at the upper GI tract. To visualize the esophagus, stomach, and duodenum, an endoscope-lit camera on the end of a tube is passed down the throat during the examination. Even with optimal PPI treatment, advanced esophagitis may be present in cases of resistant rGERD. Similarly, reflux-mediated esophageal strictures that reoccur despite prior full dilatation and optimized PPI medication should be seen as indicative of insufficiently managed acid reflux. The main purpose of upper endoscopy is to rule out non-reflux esophageal disorder and other gastric diseases, as well as to determine the presence of erosive esophagitis, which may be a sign of persistent acid reflux [9]. Kwak et al. performed an observational study on 301 rGERD and found completely normal EGD findings in 49.8% of patients and benign or incidental findings in 33.6% of patients. They concluded that endoscopy is not very useful for diagnosing rGERD because most patients have normal EGD findings and many rGERD symptoms may be due to other esophageal motility issues, non-erosive reflux disease (NERD), or healing of mucosal injury with PPI treatment. Recurrence of reflux-mediated esophageal strictures after complete dilatation and optimal PPI therapy should be interpreted as an indication of insufficiently managed acid reflux. PPI medication alone cannot cure or repair Barrett's esophageal segments; hence, the presence or persistence of Barrett's esophagus in an EGD among patients with long-term GERD exposure does not reflect evidence of resistant GERD. In this case, esophageal biopsies, if not previously done, are taken into consideration to assess for eosinophilic esophagitis, particularly if there is any indication of dysphagia [10].

Esophageal manometry or high-resolution manometry (HRM)

Esophageal HRM evaluates motor function and locates the LES for the insertion of reflux catheters; while it is not a definitive diagnostic test for GERD, it is recommended for symptomatic assessment and before invasive treatment is started. van Hoeij et al. compared HRM measurements in 69 GERD patients and 40 healthy subjects and found a significantly lower contraction amplitude (p=0.045) and basal LES pressure (p=0.034) in GERD patients than in healthy controls [11]. Finding esophageal motor disorders like diffused esophageal spasm (DES), weak peristalsis, hypertensive esophageal dysmotility, achalasia, HH, excessive upper esophageal sphincter (UES) pressure, and aberrant LES pressure is the primary goal of this procedure. The second, and perhaps more crucial, reason for esophageal manometry is to pinpoint the precise site of the LES so that reflux monitoring pH sensors can be installed. Moreover, HRM assists in determining any possible motor contraindications to normal fundoplication, such as missing contractility. HRM can measure the effects of surgical intervention and evaluate HH in addition to measuring the anti-reflux barrier using the innovative EGJ-contractile integral metric, which may be used to distinguish GERD patients from controls. Supplementary preoperative HRM protocols containing several fast swallow sequences may be considered to alter fundoplication techniques to attenuate the postoperative dysphagia risk in patients with endo flip for ambulatory reflux monitoring in rGERD [12].

Ambulatory reflux monitoring

There are two techniques to monitor esophageal reflux during on-PPI and off-PPI. When evaluating these individuals, pH monitoring can be done both on and off PPI to see if the original diagnosis was accurate. On-PPI monitoring is done to see if the symptoms are still caused by acid reflux. Regardless of whether the pH test is normal or abnormal, including a symptom-reflux correlation measure such as the symptom index (SI) and/or symptom association probability (SAP) aids in determining the relationship between acid reflux events and heartburn episodes. By assessing SI and reflux index in off-PPI patients and positive SI-RE connection seven days after PPI discontinuation, it is possible to verify the existence of pathological rGERD. The two parameters SI and SAP must be strictly connected to the symptoms and the time spent under pH 4. Hypersensitive esophageal reflux disease (HE), which is indicative of underlying visceral hypersensitivity, is defined as a positive symptom correlation with normal esophageal acid exposure. The measurement of esophageal acid exposure and the evaluation of the temporal correlation between symptoms and acid reflux events are made possible by wireless esophageal pH monitoring. Patients with rGERD are frequently evaluated using this pH monitoring method [13].

Management of rGERD symptoms

Lifestyle Modifications

Alterations in the patient's lifestyle, such as weight loss, elevating the head of the bed, and refraining from late-night eating, showed some benefits in the management of the disease in patients exhibiting resistant reflux symptoms. Avoiding habits that patients or doctors have determined to be symptom triggers is valuable for lifestyle adjustments for patients with refractory symptoms. These modifications may include following a low-bulk, low-fat diet along with eating smaller, more frequent meals. Surveys after a six-month weight reduction program in overweight and obese persons have shown that a structured weight loss program may have a greater positive influence on reflux symptoms. Improvements in reflux symptoms have been correlated with a graded response in body mass index (BMI) drop [14]. Though most studies indicate no substantial cause-and-effect link, alcohol usage should be minimized in such patients because it may aggravate esophageal symptoms. Complete dietary elimination of food groups that aggravate symptoms of GERD has not been thoroughly studied; however, avoiding trigger foods and beverages should be done individually. On the other hand, supine acid exposure can be minimized by delaying bedtime by a few hours after the last meal of the day. Sadafi et al. recruited 9631 adults aged 35-65 years in a population-based study to assess the risk factors for GERD and found the higher odds of GERD with active smoking (23% higher), alcohol intake (OR: 1.51), depression (46% higher in depressed patients), and high intake of sweets and desserts (OR: 1.02). They also found that the odds of GERD were reduced with a high intake of fiber (OR: 0.98) and dairy products (OR: 0.99) [15]. Yuan et al. recruited 1518 subjects (832 GERD and 686 non-GERD) to study the relationship of GERD with lifestyle factors. They found higher odds for GERD with fast eating (OR: 4.058) or eating hot foods (OR: 1.811), wearing corsets (2.187), high BMI (OR: 1.805), and smoking (OR: 1.521). They also showed improved outcomes with adjuvant lifestyle interventions over medication alone (p<0.001) [16].

Therapeutic Management of rGERD

Anti-secretory therapy with PPIs: The amount of time that the intragastric pH is >4.0 in the 24 hours after treatment determines how effective PPI therapy is. This should be greatest when the PPI is administered with the first meal of the day. Before assessing a PPI regimen's efficacy in treating patients with rGERD, it is imperative to verify patient adherence. Roughly half of people only use their PPI occasionally, and an astounding proportion of individuals do not take the drug as directed. Ideally, 30-60 minutes before a meal is the ideal window of time for PPI administration to prevent stomach acid output as effectively as possible. Previous studies to understand patient compliance have shown that as little as 10% of participants take their medications on time in a consistent fashion, highlighting the significance of a positive medical history. Augmenting the frequency of doses to twice a day may also be beneficial in people with severe GERD who are not responding to standard PPI therapy. This can result in additional symptomatic relief, healing of esophagitis, and a lesser acid load compared with once-daily dosing. Moving to a stronger PPI may be necessary if the optimal twice-daily dosage for 4-8 weeks is not sufficient to alleviate the symptoms or heal esophagitis. Despite the paucity of existing research, some studies indicate that PPIs have different omeprazole-equivalent potencies, while other studies support PPIs that can be used interchangeably without affecting results. Pantoprazole has a lower potency when compared to omeprazole-equivalent potencies, but rabeprazole, esomeprazole, and dexlansoprazole have higher potencies. Lansoprazole is essentially equal to omeprazole. The S-isomer of omeprazole is called esomeprazole (Nexium). In individuals with GERD and reflux esophagitis, esomeprazole is linked to greater rates of healing and symptom reduction when compared to omeprazole [17].

Long-term PPI medication generally has a greater potential benefit than the risk in patients with chronic or complex rGERD. The most frequent adverse effects are diarrhea and headache. Reduced absorption of cobalamin occurs infrequently, but a clinically relevant drop in serum B12 levels is not common. Antral G cells produce more gastrin because of PPIs' significant reduction in stomach acid output. Since the first introduction of PPIs more than 16 years ago, they have not been connected to either stomach cancer or carcinoid.

When choosing the best PPI regimen, pharmacokinetics, specifically metabolism via the hepatic cytochrome P450 (CYP2C19) system, must be considered. Fast PPI metabolization is a risk factor for rGERD in certain patients. African Americans are less likely to have CYP mutations linked to rapid metabolism, but Caucasian patients are rapid metabolizers. In contrast, individuals with Asian ancestry are more likely to have CYP mutations linked to phenotypes of normal, intermediate, and impaired metabolism. However, PPIs that avoid hepatic CYP metabolism, like rabeprazole and esomeprazole, may be used in patients who are at risk for rapid metabolism and elicit an unsatisfactory response [17].

Histamine-2 receptor antagonists (H2RA): It has been demonstrated that the duration of NAB is greatly shortened by the addition of an H2RA before bed. Patients who were still experiencing symptoms when on a normal or double-dose PPI were put on H2RAs at bedtime due to the possibility that these medications would affect the histamine-induced spike in gastric acid production that occurs at night. According to preliminary studies, the number and duration of twice-daily PPI-dose GERD patients who developed NAB were significantly reduced when H2RA was added before bedtime. On the other hand, traditional-dose and double-dose H2RA had a similar effect on NAB. Using a sample of 100 patients (58 on twice-daily PPI and 42 on twice-daily PPI+H2RA at bedtime for at least one month), Mainie et al. showed that the percentage of patients with NAB (64% versus 17%; p<0.001) was significantly decreased when a bedtime H2RA was added [18].

TLESR reducers: TLESR reducers can be considered for patients with an abnormal frequency of non-acid reflux. The drugs that can reduce the number of reflux events regardless of their acidity are theoretically desirable because of their potential use in weakly acidic or bile reflux. Since the main cause of rGERD is TLESR, baclofen helps lower the frequency and length of reflux episodes that may be causing refractory symptoms. It has been demonstrated that baclofen effectively reduces symptoms in the event of big HH. Its short half-life makes numerous daily dosages necessary for effectiveness, whereas drowsiness, dizziness, and depression of the central nervous system (CNS) limit its use. Since a variety of receptors have been demonstrated to be involved in initiating TLESR, multiple therapeutic options have evolved to treat the disorder. The two classes of drugs that exhibit the highest degree of TLESR inhibition are the metabotropic glutamate receptor 5 (mGluR5) antagonists and gamma-aminobutyric acid B (GABA-B) receptor agonists [19]. GABA-B agonist baclofen was brought to the therapeutic setting as a possible adjunctive therapy for those who did not respond to PPI therapy (once or twice daily). The medication raised the basal pressure of the LES, sped up gastric emptying, decreased reflux episodes by 43%, and decreased the TLESR rate by 40-60%. Regrettably, efforts to optimize the pharmacokinetics of alternative drugs that target the GABA-B receptor have not met anticipated results, and as of right now, no GABA-B agonist specifically targets this aspect. It has been demonstrated that baclofen greatly lessens the symptoms of duodenogastroesophageal reflux disease (DGER) and mildly affects acid reflux and DGER itself. In a study with an experimental rodent model, baclofen emerged to be useful in attenuating pain-associated responses in addition to its effect on the TLESR rate.

To counteract the negative effects of baclofen, a novel GABA-B agonist called lesogaberan with a superior CNS safety profile was developed. On a limited subset of individuals who continued to experience rGERD-related symptoms even after using PPIs, lesogaberan significantly reduced the rate of TLESRs, raised basal LES pressure, reduced esophageal acid exposure, and reduced the frequency of postprandial reflux events in this placebo-controlled, cross-over research. Additionally, it was shown that the proximal extent of gastroesophageal reflux occurrences was decreased. The frequency of reflux symptom bouts did not differ between the study's two groups, though [20].

The regulation of TLESRs has been linked to peripherally distributed glutamate receptors (mGluR5), and hence, they could be a target for GERD treatment. The only mGluR5 antagonist to be evaluated clinically was the specific negative allosteric modulator ADX10059, which showed significant reductions in TLESRs, esophageal acid exposure, and symptomatic reflux events. On the other hand, this medication was linked to a consistent increase in hepatic failure instances, abnormalities involving the CNS, and liver function tests. As a result, drug development of the ADX10059 was recently halted.

Another promising molecule that bit the dust on reaching phase 2b of the clinical trial was arbaclofen placarbil due to lack of clinical efficacy. The novel transportable pro-drug arbaclofen placarbil, also called XP19986, is the pharmacologically active R-isomer of baclofen. The drug was being investigated for the treatment of rGERD. Arbaclofen placarbil was engineered to be quickly digested to liberate R-baclofen following absorption and to be efficiently absorbed in the GI tract. Contrary to baclofen, arbaclofen placarbil absorbs efficiently from the colon, enabling the medication to be administered in a sustained-release formulation that would require fewer dose adjustments and, thus, fewer variations in plasma exposure. Consequently, a longer duration of action, subject convenience, and a better safety profile than baclofen might have resulted in possibly enhanced efficacy [19].

Invasive Methods of the Anti-reflux Management of rGERD

To provide suitable patient-specific targeted therapies, phenotypic characterization and patient counseling should be carried out if pharmacological therapy is ineffective. Patients with reflux hypersensitivity may only partially respond to surgical management, and patients with functional heartburn should not have surgery. Post the phenotypic characterization, the following surgical intervention can be considered for the planning of management of rGERD in the patient.

Laparoscopic fundoplication: Anti-reflux surgery is an option in patients who fail to respond to PPI therapy or develop complications of GERD, such as severe esophagitis, stricture, or risk of aspiration. Patients who are unable to accept or adhere to long-term medication may potentially benefit from such a surgery. Laparoscopic fundoplication is the main surgical procedure used to treat GERD. Larger peer-reviewed and controlled trials indicate that anti-reflux surgery is more beneficial for patients who have a history of partial or complete non-response to PPI medication. Patients with typical symptoms who demonstrate objective evidence of acid reflux and inadequate anti-reflux medication compliance before surgery also have better outcomes from laparoscopic fundoplication. As Nissen fundoplication is durable and effective over the long term, it is the most typical practice in the United States. The Nissen fundoplication has the best outcomes when approached laparoscopically since it offers lower perioperative morbidity without affecting clinical results. In a meta-analysis, Richter et al. included 1128 patients from seven trials to compare the effects of transoral incision-less fundoplication (TIF) and laparoscopic Nissen fundoplication (LNF) with those of sham procedures or PPIs. The authors concluded that LNF significantly improved the GERD parameters such as increased LES pressure and decreased time with pH <4 [21].

Gastric bypass: Obesity poses a challenge in refractory management. Roux-en-Y gastric bypass (RYGB) may be explored for morbidly obese patients with rGERD, despite the possibility that gastric sleeve surgery will exacerbate GERD symptoms. RYGB was linked to considerably fewer total in-hospital complications, with equivalent length of stay and risk-adjusted mortality when compared to laparoscopic fundoplication, based on a retrospective study of patients who were severely obese. In this study, it was inferred that in addition to lowering BMI and obesity-related problems, RYGB improves reflux esophagitis, lessens esophageal acid exposure for periods longer than three years, and lessens symptoms of rGERD (69.6% of the patients having preoperative GERD symptoms showed resolution). Due to the correlation between obesity and GERD, bariatric surgery is suggested for morbidly obese patients who meet the American Society for Metabolic and Bariatric Surgery (ASMBS) criteria and suffer from intolerant GERD [22]. 

LINX®: A weak sphincter is strengthened by implanting the LINX® device around the LES, which, in turn, aids in stopping the backflow of stomach contents into the esophagus. Reflux (regurgitation) and heartburn symptoms are improved among the patients if the passage of stomach content into the esophagus is restricted. While the LINX® device aids in preventing the reflux of stomach contents into the esophagus, food and liquids can still pass down the esophagus and into the stomach by causing pressure on the titanium wire while swallowing, which makes the magnetic beads to separate. The magnetic beads go back to their closed configuration once the food or drinks have entered the stomach. The FDA authorized the use of LINX® in 2012 for the treatment of severe rGERD symptoms that are non-responsive to medication regimes. Although there are only brief case studies available, LINX® has been shown in numerous prospective studies to be both safe and effective in treating individuals who are unable to control their rGERD symptoms. For the treatment of individuals with poorly controlled concurrent refractory asthma and GERD, LINX® is a feasible substitute for the Nissen fundoplication procedure as well. An increasing amount of research backs up this treatment's effectiveness, safety, and long-term viability. In a five-year follow-up study by Ganz et al., none of the patients who received a LINX® device as part of the FDA trial experienced any device migrations, erosions, or malfunctions. At five years, the patients reporting moderate to severe regurgitation decreased to 1.2% from 57% at baseline, indicating good control of rGERD symptoms [23].

EndoStim™: An electrical neuromodulator (EndoStim) with two electrodes that are put on the LES and inserted into the abdominal wall via laparoscopy has received approval in Europe and South America for esophageal neurostimulation. The device has demonstrated a significant reduction in symptom scores in rGERD patients who had poor response to PPI treatment for at least a year. Bowel perforation was one of the major adverse events linked to this technique [24]. Overall, it's still unclear if neurostimulation can effectively treat rGERD due to insufficient studies in the arena.

Stretta: Stretta increases basal pressure by applying radiofrequency energy to the LES, which alleviates rGERD symptoms. For individuals who refuse surgery (fundoplication), radiofrequency energy application to the LES (Stretta treatment) is a useful alternative. Stretta has been demonstrated to reduce refluxate volume, intraesophageal pH, and exposure of the esophagus to acid while also strengthening the anti-reflux barrier. During a 10-year follow-up, 72% of patients with rGERD demonstrated normalization of health-related quality-of-life (HRQL) ratings, and 41% reported cessation of PPI therapy completely. These results were from an open-label trial of Stretta. There is conflicting evidence, though; a recent systematic review found no discernible advantage over a placebo. Concerns of esophageal perforation previously restricted clinical use, but more recent research shows that the most frequent side effect is brief chest pain that might be mitigated without any treatment [25].

Medigus Ultrasonic Surgical Endostapler (MUSE): MUSE is a device that may be placed endoscopically and is used to create a skin flap within the esophagus, as approved by the FDA in 2014. In patients with symptomatic rGERD that are partially responsive to PPI, the MUSE device consisting of an ultrasound and a video-guided endoscopic stapler can be used. The endostapler is made up of a hard 5-cm distal portion and a long, flexible shaft that holds a staple cartridge with five titanium surgical staples. This procedure employs ultrasound and video-guided procedures with a surgical stapler to produce the fundoplication transorally. According to multicentric prospective research, 65% of patients stopped taking PPIs entirely, and 73% of patients had a substantial improvement in their GERD-HRQL score at the six-month follow-up. The percentage of patients who remained on no more than one PPI dosage per day fell from 84% to 69% during four-year follow-up, but new adverse events did not emerge. The most frequently reported adverse effects were sore throat and chest pain, which were reported by 15% and 22% of the patients, respectively. Other severe side effects reported have included bleeding, esophageal perforation, and pneumothorax with resulting empyema. However, the available clinical data is not sufficient to support routine use and needs further clinical studies to support its usage [26].

Alternative Therapies for the Treatment of GERD

Certain rGERD patients also benefit from complementary or alternative systems of medicine and therapy. Postprandial reflux episodes can be lessened and LES pressure raised with the technique of diaphragmatic breathing, as seen in a randomized trial of patients with healed esophagitis or NERD. Benefits could include a continuous improvement in quality of life and the cessation of PPIs among the patients. Acupuncture and hypnotherapy can also aid in lessening the severity of symptoms, particularly chest pain [27]. Although the precise method by which acupuncture reduces the symptoms of reflux is uncertain, theories include enhanced stomach motility and esophageal peristalsis, as well as reduced visceral hypersensitivity and LES relaxation in the patients. A randomized study comprising 30 patients who did not show improvement with standard-dose PPIs found that 10 acupuncture treatment sessions over four weeks significantly reduced acid regurgitation and daytime and nighttime heartburn [28]. In population-based research, rGERD symptoms were exacerbated by elevated psychological stress, anxiety, and/or depression; these patients may benefit from behavioral psychologist-administered tailored therapy [29].

## Conclusions

A significant portion of GERD patients struggle with rGERD. rGERD is most likely the result of many disease states rather than a single underlying cause. Medication noncompliance, visceral hypersensitivity, acid reflux, motility abnormalities, and changes in PPI metabolism are among the possible causes. It is also necessary to consider other disease states such as esophageal cancer, achalasia, and eosinophilic esophagitis. Treatment options include procedural, pharmaceutical, and non-conventional interventions. Treatment via pharmacological and surgical procedures must be tailored to the individual needs of each patient. A risk-benefit analysis of each therapeutic option should be discussed with the patient to provide better therapeutic care to everyone. Alternative therapies such as diaphragmatic breathing or yogic breathing and acupuncture may act as adjuvant treatments for the symptomatic relief and management of the disease.
